# Feedback-Related Negativity and Frontal Midline Theta Reflect Dissociable Processing of Reinforcement

**DOI:** 10.3389/fnhum.2019.00452

**Published:** 2020-01-09

**Authors:** Eric Rawls, Vladimir Miskovic, Shannin N. Moody, Yoojin Lee, Elizabeth A. Shirtcliff, Connie Lamm

**Affiliations:** ^1^Department of Psychological Sciences, University of Arkansas, Fayetteville, AR, United States; ^2^Department of Psychology, Binghamton University, State University of New York, Binghamton, NY, United States; ^3^Department of Human Development and Family Studies, Iowa State University, Ames, IA, United States

**Keywords:** prediction error, salience, reinforcement, feedback-related negativity, FRN, frontal midline theta

## Abstract

Prediction errors (PEs) encode representations of rewarding and aversive experiences and are critical to reinforcement processing. The feedback-related negativity (FRN), a component of the event-related potential (ERP) that is sensitive to valenced feedback, is believed to reflect PE signals. Reinforcement is also studied using frontal midline theta (FMΘ) activity, which peaks around the same time as the FRN and increases in response to unexpected events compared to expected events. We recorded EEG while participants completed a monetary incentive delay (MID) task that included positive reinforcement and negative reinforcement conditions with multiple levels of the outcome, as well as control conditions that had no reinforcement value. Despite the overlap of FRN and FMΘ, these measures indexed dissociable cognitive processing. The FRN was sensitive to errors in both positive and negative reinforcement but not in control conditions, while frontal theta instead was sensitive to outcomes in positive reinforcement and control conditions, but not in negative reinforcement conditions. The FRN was sensitive to the point level of feedback in both positive and negative reinforcement, while FMΘ was not influenced by the feedback point level. Results are consistent with recent results indicating that the FRN is influenced by unsigned PEs (i.e., a salience signal). In contrast, we suggest that our findings for frontal theta are consistent with hypotheses suggesting that the neural generators of FMΘ are sensitive to both negative cues and the need for control.

## Introduction

**Figure 1:**
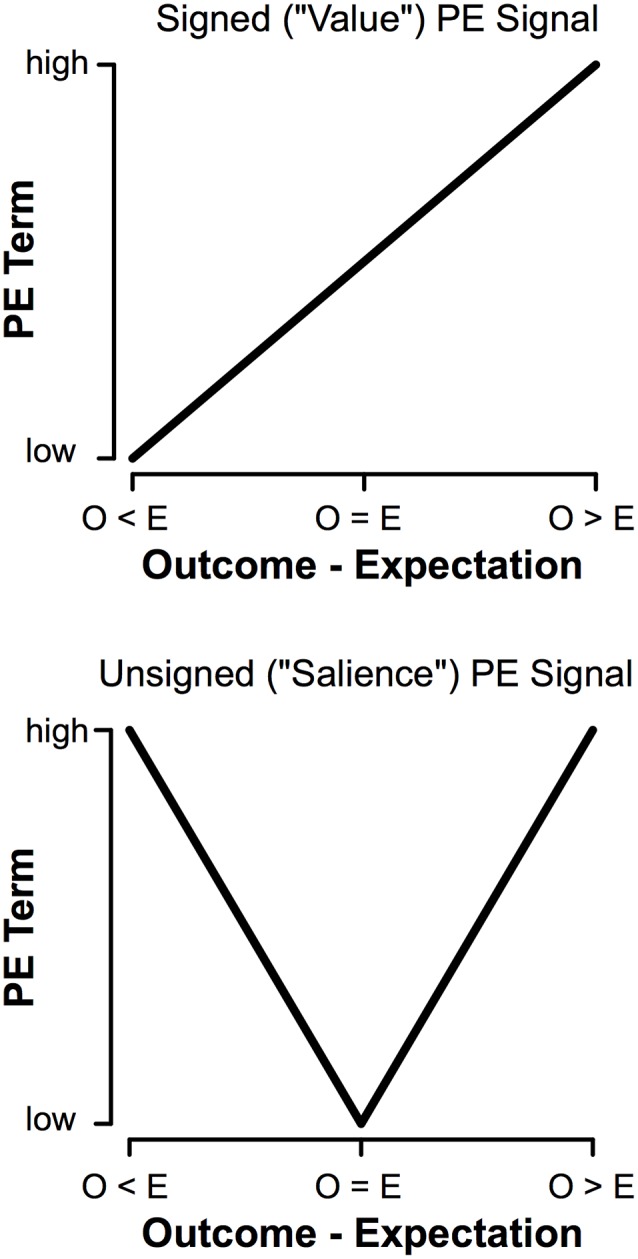
Comparison of signed (“Value”) and unsigned (“Salience”) prediction errors (PEs). The main difference is in how the terms behave when outcomes are worse than expected (O < E); a signed prediction error will decrease with worse outcomes, whereas an unsigned prediction error will scale with increasing salience regardless of the value of an outcome.

We learn the value of stimuli based on rewarding and aversive outcomes (Sutton and Barto, 1998; Dayan and Balleine, 2002). Neural representations of rewards and punishments are conveyed by prediction error signals (PEs; Schultz et al., 1997; Pessiglione et al., 2006). PEs reflect the difference between expectations and outcomes, providing a neural mechanism that optimizes behavior. PEs are signaled in two ways. Signed PEs encode outcome *value* (Rescorla and Wagner, 1972). Signed PEs increases the more rewarding an event is and decrease the more aversive an event is. In contrast, unsigned PEs encode outcome *salience* (Pearce and Hall, 1980). Unsigned PEs increase with the salience of an event, regardless of whether that event is good or bad (Figure 1). Populations of dopamine (DA) neurons encoding signed PEs (Schultz et al., 1997; Hollerman and Schultz, 1998), unsigned PEs (Fiorillo et al., 2013; Matsumoto and Hikosaka, 2009; for review see Bromberg-Martin et al., 2010), and even both (Ilango et al., 2014; for review see Schultz, 2016) have been identified, providing a neural mechanism for learning from rewarding and aversive outcomes.

The feedback-related negativity (FRN), an event-related potential (ERP) component that occurs following performance feedback, is thought to index PEs (Holroyd and Coles, 2002; Holroyd et al., 2003; Frank et al., 2005). Reinforcement Learning FRN theory (RL-FRN; Holroyd and Coles, 2002) suggests that DA projections encoding a signed PE inhibit pyramidal cells in the anterior cingulate cortex (ACC). Therefore, unexpected rewards increase the rate of DA firing, in turn reducing ACC activation. However, this hypothesis was challenged by Talmi et al. (2013), who found that the FRN not only encoded PEs in positive reinforcement but also in negative reinforcement. Talmi et al. (2012) examined the expression of signed vs. unsigned PEs in the FRN time period, failing to find a value PE but instead suggesting that the FRN resembles an unsigned PE. *Talmi’s team* recently replicated these results (Hird et al., 2018), which seemingly indicates that the FRN is consistent with an unsigned (salience) PE signal. In support of this view, Sambrook and Goslin (2015) conducted a comprehensive meta-analysis using great grand-averages, which demonstrates overlapping effects of value and salience during the FRN time period. This same team published evidence that salience signals are evident during the time period of the FRN using a single-trial approach combined with principal components analysis of the scalp ERP (Sambrook and Goslin, 2016). Sambrook and Goslin (2014) parametrically manipulated reward levels and therefore PEs and reported overlapping effects of salience and value within FRN time ranges. However, studies by this team included only positive reinforcement conditions, unlike the aforementioned studies by *Talmi’s team*. Despite recent evidence that contradicts the theory that the FRN contains a signed PE, this is far from a consensus in the field, as many authors continue to argue that the FRN reflects reward PEs (Heydari and Holroyd, 2016; Mulligan and Hajcak, 2018; Ribas-Fernandes et al., 2019). As this debate is currently ongoing and is far from resolved, the current study is informative to the current state of research on cortical correlates of reinforcement processing.

Neural activity in the theta frequency band (frontal midline theta, FMΘ) peaks during the time period of the FRN (Cavanagh and Frank, 2014) and increases in response to unexpected events (Cavanagh et al., 2012; Mas-Herrero and Marco-Pallarés, 2014). While FMΘ activation has been considered synonymous with the ERP components with which it co-occurs (Cavanagh and Frank, 2014), there is evidence that ERP and time-frequency measures index dissociable cognitive processes (Hajihosseini and Holroyd, 2013). FMΘ is related to adjustments in cognitive control, such as overriding Pavlovian biases (Cavanagh et al., 2013), but it is unclear whether FMΘ encodes a PE. Instead, FMΘ might signal the need for control as a function of surprise. This important distinction between surprise and salience is not acknowledged in much of the extant literature. Specifically, events can be surprising or unexpected without carrying any reinforcement salience, and even in paradigms that titrate wins and losses at 50%, some outcomes can be more salient (higher magnitude) than others without any element of surprise. While it has sometimes been posited that FMΘ reflects a salience PE (Cavanagh et al., 2012; Mas-Herrero and Marco-Pallarés, 2014), these results were assessed solely based on surprising outcomes in positive reinforcement and were not examined during negative reinforcement, which is a necessary condition for examination of salience PEs (Talmi et al., 2013; Huang and Yu, 2014; Hird et al., 2018). Furthermore, extant theories contend that the brain regions generating theta rhythms might be preferentially sensitive to negative, but not positive, valenced events (Cavanagh and Frank, 2014; Cavanagh and Shackman, 2015), suggesting potential differences in the signal communicated by theta rhythms depending on whether the trial cue was rewarding or aversive.

To elicit and measure reinforcing win, reinforcing loss, and non-reinforcing outcome representations in human cortex, we had participants complete a monetary incentive delay (MID) task (Knutson et al., 2000, 2001) while recording EEG. This task has been used in other ERP studies (Broyd et al., 2012), but considering the analysis of this task as a measure of PE signaling is novel. Critically, this task includes positive reinforcement conditions, negative reinforcement conditions, and control conditions, as well as multiple levels of reinforcement salience. Money was used as a reinforcer for both the positive reinforcement condition (correct responding led to increases in points) and negative reinforcement (correct responding led to avoidance of point losses). This task is different from similar designs in that it includes positive and negative reinforcement cues (signaling reinforcement *via* attaining reward or avoiding punishment) as opposed to positive and negative PEs, as utilized by Sambrook and Goslin (2014) and Talmi et al. (2012) to examine better- and worse-than-expected outcomes. In this task, we defined “unexpected” outcomes as those that are less likely at a whole-task level. This is a relatively simple definition (that more common outcomes must be more expected than less likely outcomes) utilized in the majority of ERP research that examines expectancy effects in reinforcement (for example, Talmi et al., 2012, 2013; Hajihosseini and Holroyd, 2013; Huang and Yu, 2014). At a task-averaged level, this definition holds (that more common outcomes are more expected than less common outcomes), but it is likely that at a trial-by-trial level individual expectancies are influenced by factors other than mere probability (for example see Fischer and Ullsperger, 2013). While it is likely that trial-by-trial approaches could more accurately model shifts in expectancies compared to the one-size-fits-all approach of saying more common events are “expected” and less common events are “unexpected,” we chose to use the standard (task-averaged) approach in this analysis to facilitate comparison of our results with prior FRN analyses.

Our task improves on previous designs used for studying positive and negative reinforcement since direct comparison of positive and negative reinforcement conditions is generally not permissible because rewards and aversive stimuli are presented in different modalities (money and electrical shock; Talmi et al., 2013; Heydari and Holroyd, 2016). This introduces an experimental confound—specifically, the punishment is delivered immediately, while the reward is delivered after the experiment. By using points (money) as a reinforcer for both positive and negative reinforcement conditions, our results control for the potential confound of reinforcement delivery timing. Furthermore, the modified MID task included a condition that did not involve reinforcement (a control condition). This manipulation allowed us to test assumptions that previous studies have been unable to test in the absence of a non-reinforced control condition. Specifically, a PE signal must reflect outcome information for both positive and negative reinforcement, but not for conditions that do not involve reinforcement, while an uncertainty signal should be sensitive to unexpected outcomes with no reinforcement value (control conditions).

We hypothesized that FRN amplitude would be sensitive to salience PEs, rather than value PEs. We designed our MID task so that the timing was titrated to give a 66% accuracy rate (high expectation of getting a win). Therefore, we hypothesized that FMΘ activation would be greater for error feedback compared with correct feedback in both reinforcing (negative and positive) and control trials, consistent with the idea that FMΘ reflects unexpectedness regardless of reinforcement salience.

## Materials and Methods

### Participants

The sample consisted of 81 undergraduate students at the University of New Orleans. Participants were excluded from the study if they had a current psychiatric diagnosis, were currently using any psychoactive medication or had uncorrected visual impairments. Additionally, five participants were excluded due to poor EEG connection (more than 10 bad electrodes) and 25 participants were excluded due to insufficient trial count, as detailed in the processing section. The final sample size consisted of 51 (26 female; 7 left-handed; age mean = 21, age SD = 4) participants. Excluded participants did not differ from those included in terms of sex $\chi_{\left(1,N=81\right)}^{2}$ = 1.46, Fisher’s exact *p* = 0.25, age, *t*_(63)_ = −1.41, *p* = 0.16, or handedness, $\chi_{\left(1,N=69\right)}^{2}$ = 0.87, Fisher’s exact *p* = 0.37. Students were paid for their participation based on how many points they accrued during the behavioral task. Informed consent was obtained from all participants prior to beginning data collection. All components of this study were approved by the University of New Orleans Institutional Review Board.

### Procedure

Participants were introduced to the testing environment and informed consent was obtained. Participants completed a battery of self-report measures before being seated 67 cm in front of a computer monitor. The EEG net was applied, and the participant completed a set of practice trials. If their accuracy was below 66% they repeated the practice block, thus ensuring that each person fully understood the task (only one person had to repeat the practice block). Following the practice block, the participants completed the behavioral task (detailed below) while EEG was recorded.

### Task Design

Participants completed a modified MID task (Knutson et al., 2000, 2001). An EEG compatible version of this task is described by Broyd et al. (2012). We used white stimuli presented on a black background on a 17-inch monitor using E-prime software version 2 (Psychology Software Tools, Inc., Pittsburgh, PA, USA) for all task stimuli.

The MID task begins each trial by presentation of one of three white cue stimuli. This cue indicated the trial type. Specifically, a circle indicated positive anticipation, a square indicated negative anticipation, and a triangle indicated no reinforcement anticipation (i.e., the control condition). Point level was signified by the lines on the cue—one line for low point level trials (±5 points), two lines for medium point level trials (±10 points), and three lines for high point level trials (±20 points). Triangle (control) cues did not have point level indicators. The cue was on-screen for 250 ms and was followed by a fixation for 1,000–1,500 ms (jittered).

Following the cue, a white square target was shown that required subjects to press a button quickly in order to obtain a favorable outcome. Participants were instructed to respond as quickly as possible when they saw the target stimulus. Responses were made using the thumb of the dominant hand using a button box, and the criteria for logging a response as correct or incorrect was whether the response occurred within an allotted time period. The length of the stimulus-response window was dynamically adjusted to maintain a performance accuracy of 66%. The titration was accomplished by lengthening the time participants were allowed for responses by 20 ms anytime overall accuracy was below 66% and shortening the time allowed for responses by 20 ms any time overall accuracy was greater than 66%.

**Figure 2:**
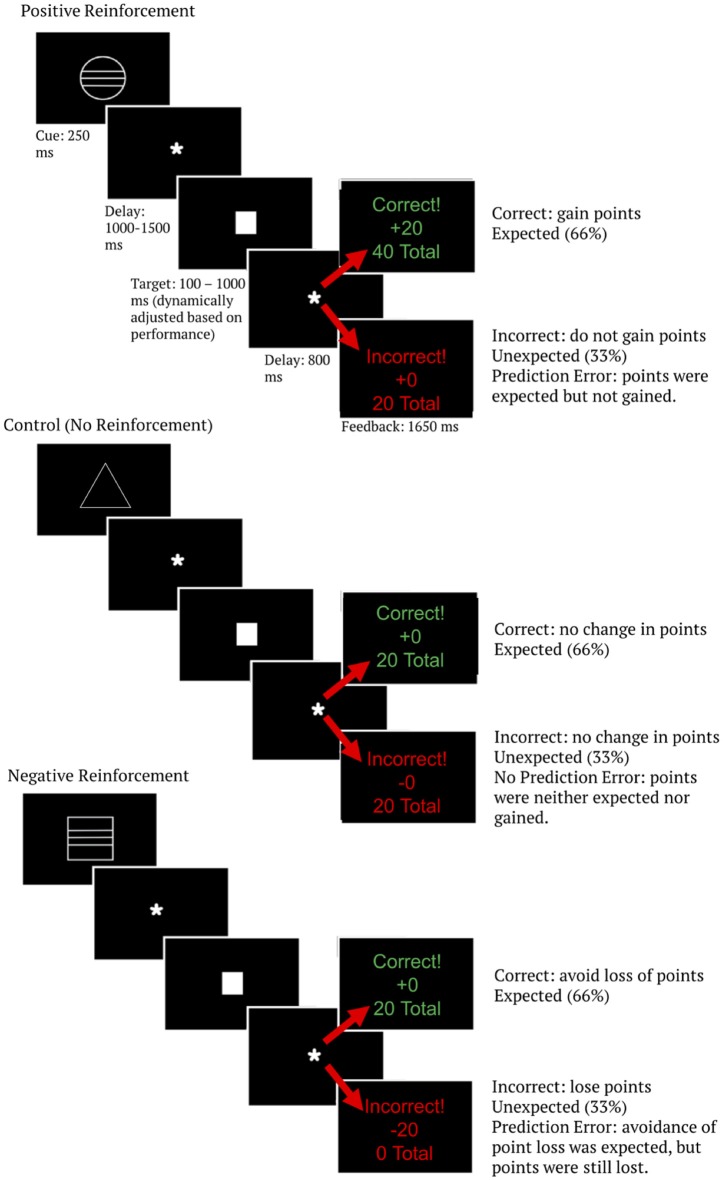
Monetary incentive delay (MID) task diagram. Image is scaled up for readability. The total number of points shown as the last line of feedback represents a running average over the entire task, allowing participants to track their progress as the task went on.

Following the response period subjects were given outcome feedback, which consisted of the word “correct” or “incorrect” on the top line, the outcome of the trial on the middle line (representing how many points were gained or lost on that trial), and the total score over all trials on the bottom line. Because wins were titrated at 66%, this task created a positive expectation. That is, the expectation was of gaining a reward (positive reinforcement; gaining points) or avoiding an aversive stimulus (negative reinforcement; avoiding the loss of points) for correct trials. This task contained 630 trials across three blocks. The middle block was a social competition manipulation that was designed to address a different research question (Lee, unpublished manuscript) and was not analyzed here, resulting in the final analysis consisting of 420 trials. See Figure 2 for a diagram of the modified MID task. Behavioral results from this task are analyzed and described in the supplementary materials (Supplementary Figure S1). Importantly, accuracy did not differ between reinforcement type or point levels (given the task speed titration to show a 66% accuracy rate, this is not surprising). Therefore, the expectancy of a win also did not differ between reinforcement type or point level conditions and thus is not an explanation for reinforcement type or point level condition differences in brain data.

### EEG Data Collection and Analysis

#### EEG Data Collection and Processing

Dense-array EEG was sampled at 250 Hz using a 129-channel GSN Hydrocel EEG net and Netstation software (Electrical Geodesics Inc., Eugene, OR, USA). Once impedance for all channels was reduced to below 50 kΩ, data acquisition began. All channels were referenced to Cz during recording. Data were processed using EEGLAB 13 (Delorme and Makeig, 2004) running in MATLAB 2016b (Mathworks). Continuous data were high-pass filtered using an 826-point zero-phase FIR filter with a cutoff frequency of 0.5 Hz (Winkler et al., 2015), and low-pass filtered using an 82-point zero-phase FIR filter with a cutoff frequency of 50 Hz. Filtering was achieved using the EEGLAB pop_eegfiltnew function, which calls the MATLAB filtfilt function. Bad channels were detected and removed using EEGLAB’s clean_rawdata plugin (Kothe and Makeig, 2013; Bigdely-Shamlo et al., 2015; Mullen et al., 2015). Using this tool, channels were removed if they were flat for more than 5 s or had correlations lower than 0.7 with their robust estimate (calculated using neighboring channels). The average number of bad channels detected was 5.09 ± 3.66 (range: 0–21). Subjects with greater than 10 bad channels (*n* = 5) were excluded from further analysis. In order to accommodate edge artifacts resulting from time-frequency convolution, continuous EEG data were time-locked to the appearance of feedback and epoched into relatively long windows extending from 2 s before feedback onset to 2.5 s after feedback onset. Epochs were removed based on a lax threshold of ±500 μV, thus removing epochs containing extreme artifacts which can be problematic for ICA processing but preserving ocular and other stereotyped artifacts (Delorme and Makeig, 2004; Jung et al., 2000a,b). Infomax independent component analysis (ICA; Makeig et al., 1996) was computed, and independent components representing ocular, muscle, or other stereotyped artifacts were detected and removed using the ADJUST plugin for EEGLAB (Mognon et al., 2011). While ADJUST shows high sensitivity for the removal of eye artifacts (Mognon et al., 2011), we nevertheless verified the performance of this algorithm by manual examination of remaining ICA components and continuous EEG to ensure no blink or eye movement artifacts remained. Since ICA can only detect and remove stereotyped artifacts (Jung et al., 2000a,b), remaining artifacts were removed *via* thresholding individual epochs at ±200 μV. Finally, removed channels were interpolated spherically and the EEG was re-referenced to the montage average. Subjects were excluded from analysis if they had fewer than 10 trials of clean data in any condition (excluded *n* = 25), following the method used in the original manuscript describing this task design (Broyd et al., 2012). The average number of removed epochs for the included sample (after participant exclusion) was 36.33 ± 39.24 out of 420 total (8.9% of data epochs were removed in the included sample).

#### EEG Analyses

All ERP analyses were conducted at electrode FCz where FRN amplitude was maximal, in line with typical FRN studies (Donkers et al., 2005; Cohen and Ranganath, 2007; Broyd et al., 2012; Hajihosseini and Holroyd, 2013). ERP waveforms were baseline corrected for 300 ms preceding the feedback stimulus. Based on the inspection of scalp grand-averaged topographic plots and ERP waveforms, FRN activation peaked at 300 ms and was therefore calculated as the mean voltage deflection between 250–350 ms. We quantified FRN activity as the mean activity in each condition from 250 to 350 ms since mean windows are less likely to contain artifact compared to minimum measures or base-to-peak measures (Luck, 2014; Luck and Gaspelin, 2017).

Time-frequency transformations were calculated using the EEG lab newtimef() function. Time-domain data were transformed to a time-frequency representation by convolving single trials with a family of Morlet wavelets (Goupillaud et al., 1984) centered on 30 logarithmically-spaced peak frequencies ranging from 1 Hz to 30 Hz. The number of cycles used for each wavelet was adaptive, ranging from three cycles at 1 Hz to 15 cycles at 30 Hz. The single-trial spectral power was taken as the squared magnitude of the complex result of the convolution [(real^2^) + (imaginary^2^)]. Trial-averaged power values were normalized using a decibel (dB) transform [dB = 10 × log10(power/baseline)] within each frequency band, where the baseline was the average power at each peak frequency band from 500 to 200 ms before feedback. Baseline correction was done using a condition-averaged baseline. This baseline normalization is necessary for condition comparisons, as it represents data from all conditions, participants, sensors, and frequencies on the same scale, thus allowing quantitative comparison (as outlined in Cohen, 2014). Inspection of grand-average time-frequency plots showed that FMΘ activation was maximally centered on electrode FCz between 200 and 450 ms post-stimulus (Supplementary Figure S4), a time range and topography that was consistent with previous studies (Luu et al., 2004; Hajihosseini and Holroyd, 2013). Therefore, FMΘ activation was also measured at electrode FCz. Based on inspection of the grand-average spectrogram, FMΘ activation was measured as the mean activation between 4 and 8 Hz (exact frequencies: 4.09, 4.59, 5.17, 5.81, 6.53, and 7.34 Hz) and from 200 to 450 ms. Cohen and Donner (2013) argue that conflict-related theta might be non-phase-locked in nature, and Cohen (2014) suggests that one way to examine whether the calculated theta activity is phase-locked or non-phase-locked is to subtract the averaged ERP from single trials. However, this procedure, as far as we are aware, has never been used to examine reinforcement-related theta activity. We present an exploratory analysis of non-phase-locked theta activity in the supplement (Supplementary Figure S3) but present the more standard full time-frequency data in the main body of the manuscript to facilitate comparison with current literature.

While frequencies other than theta were not a focus of the current analysis, time-frequency plots at FCz showed striking task-related influences at alpha frequencies (9–15 Hz). Therefore, we present an exploratory analysis of the alpha activity in the supplement (Supplementary Figure S2). Note that while we decided to include this analysis *post hoc*, we chose the time and frequency windows for which alpha was extracted by examining condition-averaged power so as not to bias our results. For alpha analysis, we measured activation between 9 and 15 Hz (exact frequencies: 9.28, 10.44, 11.74, 13.20, and 14.84 Hz) and from 300 to 600 ms post-feedback. Similarly, delta activation has been implicated in reinforcement, particularly reward processing (Bernat et al., 2015). We extracted delta activity from sensor Pz with a frequency range of 1–3 Hz (exact frequencies: 1, 1.12, 1.26, 1.42, 1.60, 1.80, 2.02, 2.27, 2.56, and 2.87 Hz) and a time range of 300–600 ms based on examination of condition-averaged activity. Results of delta and alpha time-frequency analysis are described in the supplement (Supplementary Figure S2).

#### Statistical Analysis

To determine if there were any FRN or FMΘ differences due to reinforcement type, outcome, or point level of trial, two 2 (positive reinforcement or negative reinforcement) × 2 (correct or error) × 3 (low, medium, or high point level) repeated-measures ANOVA were conducted on FRN and FMΘ activation. However, since the control condition (triangle, no reinforcement value) did not include multiple point levels, two separate 3 (positive, negative, or no reinforcement) × 2 (correct or error) repeated-measures ANOVAs were conducted on FRN and FMΘ activation. The three-point levels were averaged over for these analyses for both positive and negative reinforcement since control conditions did not include multiple point levels. Repeated-measures ANOVAs were conducted in SPSS and the Greenhouse–Geisser correction was applied to all repeated-measures tests with more than one degree of freedom. Significant omnibus main effects and interaction effects from ANOVAs were characterized *post hoc* using the EMMEANS command in SPSS (Field, 2013). Since the ANOVA models did not include any covariates and no cells were missing from the analysis (i.e., all participants completed all conditions), these comparisons are equivalent to simple linear contrasts. For all *post hoc* contrasts of significant *F*-tests, we used a Bonferroni correction to control for multiple comparisons. Full output of all ANOVAs and post hoc contrasts are presented in Supplementary Tables S1–S12.

We also ran control analyses to demonstrate that activation during the baseline window did not differ by condition. These analyses were necessary because two of the main factors of interest (reinforcement type and point level) were already known to the participant at the time of feedback, and this knowledge could have led to condition differences in the baseline window which may have spilled over into the effects of interest (Cohen, 2014). For theta baseline analysis, we computed raw spectral power (no baseline correction) for each level of reinforcement type and point value and averaged spectral power over theta frequencies from −500 to −200 ms pre-feedback. For ERP baseline analysis, we averaged the evoked potential from −300 to 0 ms pre-feedback for each level of reinforcement type and point level. For baseline analysis, we averaged over, correct and error feedback within each level of reinforcement type and point level, as these conditions were not yet known to the participant before feedback. We conducted a repeated-measures ANOVA with one factor (trial type) consisting of seven levels (positive reinforcement—large, positive reinforcement—medium, positive reinforcement—small, negative reinforcement—large, negative reinforcement—medium, negative reinforcement—small, and control) to determine whether baseline theta power or ERP differences existed for any conditions.

## Results

### Condition Differences in Feedback-Related Negativity

**Figure 3:**
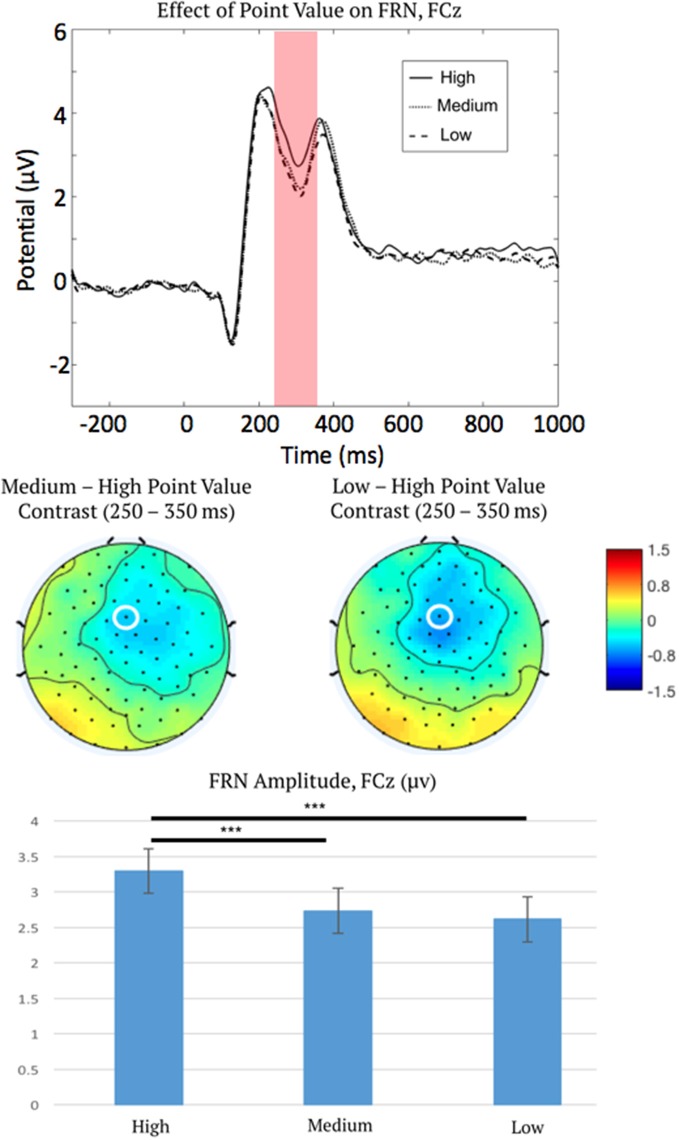
Event-related potential (ERP) waveforms measured at FCz (top), topographic distributions of the medium-high and low-high difference in the feedback-related negativity (FRN) time window (middle), and mean FRN amplitudes (bottom), showing the main effect of point salience on the FRN. This plot describes the main effect of point level as confirmed *via* ANOVA, therefore all ERP waveforms are collapsed over the correct and error outcomes, and positive and negative reinforcement. Note ERPs are plotted negative downward, such that larger FRN activation is depicted as a waveform drop. FCz is marked by a white ellipse. The shaded region is the time period across which the FRN was measured. Error bars represent 1 ± SEM. ***p < 0.001.

Preliminary analysis demonstrated that there were no detectable condition differences in the ERP baseline window of −300 to 0 ms pre-feedback, *F*_(6,45)_ = 1.18, *p* = 0.32, *η*^2^ = 0.02, ε = 0.9. Therefore, we carried out all statistical testing of FRN activation using this choice of baseline window. The initial three-way repeated measures ANOVA on FRN amplitude indicated a main effect of point level (low, medium, or high) on FRN amplitude, *F*_(2,100)_ = 11.52, *p* < 0.001, *η*^2^ = 0.19, ε = 0.92 (Figure 3), as well as an interaction between reinforcement type (positive or negative reinforcement) and outcome (correct or error), *F*_(1,50)_ = 50.50, *p* < 0.001, *η*^2^ = 0.50, ε = 1.0.

To characterize the main effect of point level (high vs. medium vs. low point-level; averaging over the other factors not included in this effect), *post hoc* linear contrasts were examined as described in “Statistical Analysis” section. Contrasts indicated that FRNs were less negative for high-point trials than both medium-point trials, mean difference = 0.56 μV, 95% CI = (0.3, 0.82), *p* < 0.001, and low-point trials, mean difference = 0.68 μV, 95% CI = (0.34, 1.02), *p* < 0.001. The FRN difference between medium and low point trials was not significant.

*Post hoc* linear contrasts (“Statistical Analysis” section) was also used to characterize the significant interaction between reinforcement type and trial outcome (averaging over point level). These tests, therefore, examined the simple effect of reinforcement type (positive or negative) on FRN amplitudes within each level of outcome (correct or error), as well as the effect of the outcome on FRN amplitudes within each level of reinforcement type. Interestingly, results revealed opposite effects between positive and negative reinforcement. For positive reinforcement conditions, FRN amplitudes were more negative for reward omission (error trials, do not gain points) compared to reward delivery (correct trials, gain points) feedback, mean difference = −0.70 μV, 95% CI = (−1.14, −0.26), *p* = 0.002. However, for negative reinforcement conditions, FRN amplitudes were more negative for avoidance of aversive stimuli (correct trials, avoid losing points) compared to aversive stimulus delivery (error trials, lose points), mean difference = 1.01 μV, 95% CI = (0.58, 1.43), *p* < 0.001.

We interpret this finding as support for salience coding by FRN activation because the salience of point feedback (level of points), regardless of signed value (positive or negative reinforcement), impacts FRN amplitude (i.e., the ANOVA indicated a significant main effect of point level rather than an interaction between point level and reinforcement type). FRN amplitude did not differ in sign between positive and negative reinforcement but instead coded the absolute value of the delivered stimulus (FRN became less negative with increased point level for both positive and negative reinforcement; see Figure 3, bottom panel).

For the second ANOVA on FRN amplitude, point level (low, medium, or high) for positive and negative reinforcement was averaged over in order to allow comparison with control conditions, which did not include multiple point levels. The two-way repeated measures ANOVA indicated a main effect of reinforcement type (positive, negative, or control) on FRN amplitude, *F*_(2,100)_ = 22.88, *p* < 0.001, *η*^2^ = 0.31, ε = 0.73, which was subsumed by a significant interaction between reinforcement type and outcome (correct or error), *F*_(2,100)_ = 17.14, *p* < 0.001, *η*^2^ = 0.26, ε = 0.90.

**Figure 4:**
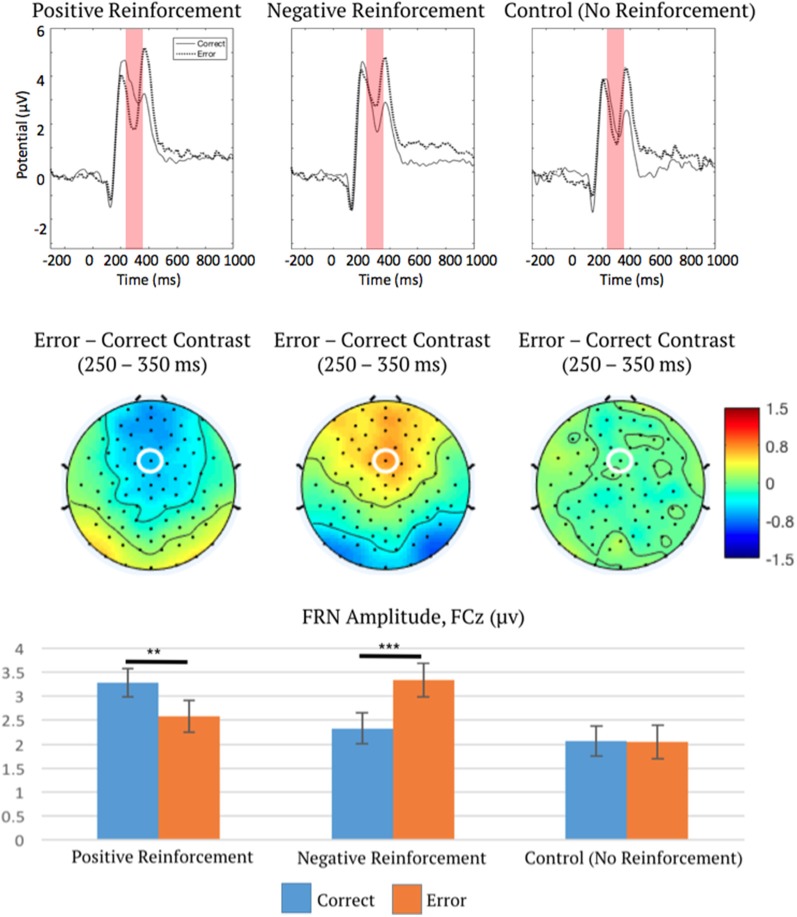
ERP waveforms measured at FCz (top), topographic distributions of the error-correct difference in the FRN time window (middle), and mean FRN amplitudes (bottom), depicting interaction effect of reinforcement type and outcome on FRN. This plot describes the interaction effect of reinforcement type and outcome as confirmed *via* ANOVA, therefore all ERP waveforms are collapsed over low, medium, and high point levels. Note ERPs are plotted negative downward, such that larger FRN activation is depicted as a waveform drop. FCz is marked by a white ellipse. The shaded region is the time period across which the FRN was measured. Error bars represent 1 ± SEM. **p < 0.01, ***p < 0.001.

*Post hoc* linear contrasts (“Statistical Analysis” section) were used to characterize the interaction of reinforcement type (positive, negative, or control) and outcome (correct or error) on FRN amplitude. Critically, in control trials there was no effect of outcome (correct or error) on FRN amplitude (*p* = 0.94), unlike FRN amplitudes in positive (*p* < 0.01) and negative (*p* < 0.001) reinforcement conditions (Figure 4). This important control analysis shows that FRN activation is only sensitive to reinforcing error feedback, but not to non-reinforcing error feedback. Furthermore, while correct and error outcomes were delivered at unequal rates in this study, this second analysis indicates that in the absence of reinforcement, expectancy differences in correct and error rates do not impact FRN amplitude. This supports the previous theory that the FRN signals a PE, but our results refine this theory and suggest that neural activation during the FRN time period follows a salience function rather than a value function.

### Condition Differences in Frontal Midline Theta

Preliminary analysis of non-baseline-corrected time-frequency power demonstrated that there were no detectable condition differences in the baseline window of −500 to −200 ms pre-feedback, *F*_(6,45)_ = 0.96, *p* = 0.44, *η*^2^ = 0.02, ε = 0.71. Therefore, we carried out all statistical testing of theta activation using this choice of baseline window. All FMΘ analyses were conducted identically to the FRN analyses described already. The initial three-way repeated-measures ANOVA showed no effect of point level (low, medium, or high) on FMΘ amplitude, *F*_(1,100)_ = 2.30, *p* = 0.11, *η*^2^ = 0.04, ε = 0.99, and no interaction of point level with any other variables, all *p* > 0.1. These null results rule out point level as an explanation for any differences in theta activation. Therefore, low, medium, and high point level trials were averaged over for further theta analyses.

A second two-way repeated measures ANOVA on FMΘ power indicated a main effect of outcome (correct or error), *F*_(1,50)_ = 17.14, *p* < 0.001, *η*^2^ = 0.26, ε = 1.0, which was subsumed by a significant interaction between reinforcement type (positive, negative, or control) and outcome (correct or error), *F*_(2,100)_ = 4.64, *p* = 0.016, *η*^2^ = 0.09, ε = 0.85.

**Figure 5:**
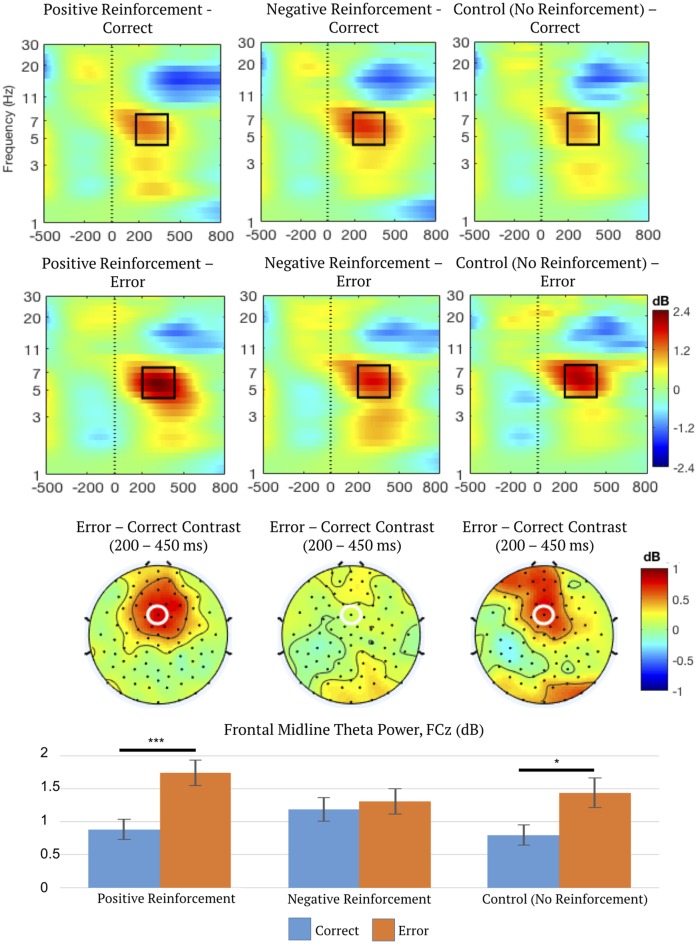
Time-frequency spectrograms measured at FCz (top), topographic distributions of the error-correct difference in frontal midline theta (FMΘ) time (200–450 ms) and frequency ranges (4–8 Hz; middle), and mean FMΘ power (bottom) depicting the interaction between reinforcement type (positive, negative, or control) and outcome (correct or error). Frequency-specific power is dB-transformed using −500 to −200 ms pre-feedback as baseline. FCz is marked by a white ellipse. Black outlines enclose the region of data exported for statistical analysis. Error bars represent 1 ± SEM. *p < 0.05, ***p < 0.001.

*Post hoc* linear contrasts (“Statistical Analysis” section) were used to characterize the significant interaction between reinforcement type and outcome. The results indicated that error trials resulted in greater theta activation than correct trials for positive reinforcement, mean difference = 0.86 dB, 95% CI = (0.53, 1.19), *p* < 0.001, and control trials, mean difference = 0.64 dB, 95% CI = (0.13, 1.15), *p* = 0.016. However, *post hoc* linear contrasts indicated no effect of outcome (correct or error) in negative reinforcement trials suggesting that in this paradigm theta activation did not differentiate between avoided (correct, avoidance of loss of points) and delivered aversive (loss of points) stimuli. See Figure 5 for a summary of FMΘ results.

For correct feedback, FMΘ power was greater for negative reinforcement than positive, mean difference = 0.30 dB, 95% CI = (0.04, 0.57), *p* = 0.021. This pattern was reversed for error feedback; FMΘ power was greater for positive reinforcement than negative, mean difference = 0.44 dB, 95% CI = (0.08, 0.79), *p* = 0.013. Therefore, in negative reinforcement conditions, FMΘ power is elevated even for correct feedback, compared to both correct positive and correct control conditions, but is not sensitive to error feedback. This supports the theory that overlapping regions in the midcingulate cortex index negative affect and cognitive control signaling (Shackman et al., 2011), and supports the hypothesis that FMΘ incorporates elements of both cognitive control and negative cues. FMΘ might index overlapping information about negative affect and unexpectedness, but does not always appear to signal PEs—evidenced by the significant effect of error feedback on FMΘ in control conditions. It is important to note in this case that unsigned PEs and unexpectedness are not one and the same, although some authors have suggested they are. As mentioned previously, outcomes with no reinforcement salience can still be “surprising” while equally expected outcomes can be more or less salient than one another. More specifically, a PE incorporates information about expectancy and outcome valence, not merely surprise. Therefore, this might support the notion that theta signals surprise but not necessarily unsigned PEs.

## Discussion

### The Reinforcement Learning FRN Theory (RL-FRN)

RL-FRN (Holroyd and Coles, 2002) specifies that the FRN reflects a signed PE. RL-FRN suggests that DA neurons project to the ACC, inhibiting pyramidal cells. This inhibitory function accounts for decreased FRN amplitudes during reward omission compared to reward delivery. Reward omission could decrease DA activity from baseline, disinhibiting pyramidal cells in ACC and producing a more negative FRN in comparison to reward delivery. In our study, the control condition did not show a difference between correct and error trial outcome. This suggests that the FRN is reinforcement-specific, in line with the RL-FRN theory. While it is possible that the FRN results are contaminated by component overlap with the P3, as in Novak and Foti (2015), this is unlikely given the topographic maps of the FRN effect. More specifically, this effect is clearly mediofrontal in both positive and negative reinforcement conditions, suggesting that this signal is not contaminated by the P3 component overlap.

Our study included negative reinforcement conditions, in addition to the more common positive reinforcement conditions. In negative reinforcement conditions, a correct response resulted in the avoidance of an aversive stimulus (avoiding loss of points or money). Meanwhile, if subjects were too slow in their response an aversive stimulus was delivered (i.e., loss of points or money). Critically, FRN amplitude was more negative for the avoidance of an aversive stimulus than for the delivery of an aversive stimulus. These results follow those of recent studies in suggesting that the FRN might encode unsigned (salience) PEs rather than signed (value) PEs. Talmi et al. (2013) alternated blocks of positive and negative reinforcement and found that FRN amplitudes were larger (more negative) for omitted rewards compared to delivered rewards, as expected. Talmi et al. (2013) also found larger FRN amplitudes for avoided aversive stimuli than for delivered aversive stimuli. Similarly, Huang and Yu (2014) used a blocked presentation of positive and negative reinforcement trials and found larger FRN activation for the avoidance of loss than for losses. These results mirror ours and suggest that the FRN encodes salience PEs rather than value PEs. Notably, a 2015 review by Sambrook and Goslin used great grand-averages to conduct a meta-analysis of ERP studies on reward PEs. This study found evidence that the FRN encoded information in a manner consistent with a reward PE, but this study also found notable effects of salience throughout many parts of the waveform. This raises the important need to examine under which conditions the FRN behaves as a signed or unsigned PE.

Heydari and Holroyd (2016) used a difference wave approach and found an apparently delayed reward positivity in negative reinforcement, occurring during the time period of the P3 rather than the FRN. Our results instead indicate that the mediofrontal topography of the FRN is reversed for negative compared to positive reinforcement—i.e., all “salient” outcomes (point gains and point losses) appear to inhibit the FRN. A possible explanation for the delayed signal found in Heydari and Holroyd (2016) is that they used different types of reinforcers for positive compared with negative reinforcement conditions. Since money (positive reinforcer) was paid at the end of the task but electrical shock (negative reinforcer) was delivered immediately, this introduces an experimental confound in the timing of reinforcement delivery. The MID task titrates wins and losses at different rates (66% and 33% loss), and therefore it is possible that this result is skewed by the different expectancies for different valenced outcomes. This possibility is reduced through the analysis of the control condition, where results indicate that different expectation rates for wins or losses do not impact the FRN, in the absence of reinforcing feedback. Still, we are unable to make concrete claims as to whether the expectancy difference between win and loss outcomes might have contributed to the apparent FRN differences we observed. However, von Borries et al. (2013) note that FRN amplitude depends on outcome valence but is insensitive to expectancy. A similar effect was also reported by Hajihosseini and Holroyd (2013), whose results indicated that the FRN was sensitive to outcome valence over and above any explanatory power of expectancy.

The idea that the FRN encodes salience PEs for poor unexpected outcomes supports our finding of more negative FRNs for omitted rewards and losses compared to delivered rewards and losses, as well as our lack of FRN differences in control conditions. Furthermore, we found the main effect of point level (low, medium, or high) on the FRN, i.e., for both positive and negative reinforcement, FRNs were less negative for high point levels than low point levels. Since point level is positively valenced for positive reinforcement conditions (references gains in points) and negatively valenced for negative reinforcement conditions (references loss of points), our main effect of point level provides further evidence that the FRN signals salience, not value. However, this study is unable to authoritatively describe whether these signals are due to “bad” outcomes or to unexpected outcomes. Future studies should examine modification of this task where all conditions analyzed are equally likely, or where probability is parametrically modified, to further dissociate the information representation of the FRN. Furthermore, in this task “unexpected” outcomes were determined simply to be those outcomes with lower probability (error outcomes). While this must hold true for the condition averages across the entire task (more common outcomes are by definition expected in comparison to less common outcomes), this definition does not necessarily hold at a trial-by-trial level. That is, it is likely that the expectancy of an outcome at a trial-by-trial level is influenced by a weighted average of previous outcomes, as well as influenced by the participant’s subjective reading of their own behavior. While it is impossible to know what a participant subjectively believes about their own behavior, future analyses should use fine-grained trial-by-trial analysis to disambiguate the exact representation of PE and reinforcement.

### Frontal Midline Theta Is Not Reinforcement-Specific

We observed increased theta power in response to unexpected outcomes (i.e., error trials, unexpected because the task was weighted towards correct trials, 34% vs. 66%) in positive reinforcement conditions and control conditions. This finding is inconsistent with the notion that FMΘ indexes a PE and highlighting the importance of including a control condition where subjects are primed with a point level of zero. Interestingly, in negative reinforcement conditions, the outcome did not influence FMΘ power. This finding is consistent with the hypothesis that frontal theta is sensitive to surprise, the need for control, and negative cueing (Cavanagh and Shackman, 2015). FMΘ is prominent following the need for control, as in error monitoring. However, our results indicate that FMΘ is overall enhanced following negative cues, even in correct feedback conditions. Since negative reinforcement/correct theta is enhanced over positive reinforcement/correct theta, we conclude that theta is overall enhanced following the cueing of negative reinforcement. Specifically, following correct feedback FMΘ is elevated for negative reinforcement conditions compared to positive reinforcement, which might represent an effect of aversive priming on theta signaling. However, theta is decreased for error feedback in negative reinforcement conditions compared to positive reinforcement error feedback—this suggests that negative priming might reduce subsequent control abilities. Probably the potential for loss of points resulted in increased baseline FMΘ power, such that the increase in power for error feedback was not significant. These results are broadly consistent with the theory that FMΘ acts as a general “alarm” signaling the need for increased control and negative cueing (Cavanagh and Frank, 2014). Although not a focus of the current study, alpha frequencies and delta frequencies were also examined. Alpha and delta frequencies showed a power increase for error outcomes compared to correct outcomes (S1), suggesting that neural oscillatory responses might be tightly linked to outcome processing but not reinforcement.

## Conclusion

Our findings are consistent with the hypothesis that the FRN signals PEs while FMΘ power instead might reflect a compound signal of control and aversion, but does not index PEs, in line with the Adaptive Control Hypothesis (tACH; Cavanagh and Shackman, 2015). Critically, we show that the FRN is consistent with an unsigned (salience) PE signal, rather than a signed (value) PE signal, which is not in accordance with the RL-FRN theory. This study uses the MID task, and therefore is unable to directly test for expectancy. We note that while losses are unexpected in control conditions, there is no impact of loss on the control FRN, which is in line with previous literature suggesting that outcome valence impacts the FRN over and above expectancy (Hajihosseini and Holroyd, 2013; von Borries et al., 2013). These results add to an overall understanding of the diversity of frontal cortical function during reinforcement. While in this particular paradigm the FRN is closer to a salience than value signal (as indicated by the polarity flip between positive and negative conditions), evidence suggests that the representation of a value or salience signal in the FRN is highly context-dependent (Sambrook and Goslin, 2014, 2015). Finally, previous studies have shown context-dependence of the FRN such that expectations are modulated by previous outcomes using blocked presentation designs. Our design instead randomly intermixed positive, negative, and control trials (with fully predictive cues so the subject knew which trial type they were on). Since the probability of a previous trial being a positive or negative reinforcement one was equal, we argue that context-dependence is unlikely to influence our results. Stated differently, other than the cue, the expectancy for positive, negative, and control trials must be equivalent due to the random design. We conclude that in the context of an established electrophysiological paradigm examining reinforcement processing (Broyd et al., 2012), the FRN encodes salience through similar mechanisms in negative reinforcement and positive reinforcement, providing a common pathway for reinforcement through signaling unsigned PEs.

## Data Availability Statement

The datasets generated for this study are available on request to the corresponding author.

## Ethics Statement

The studies involving human participants were reviewed and approved by University of New Orleans Institutional Review Board. The patients/participants provided their written informed consent to participate in this study.

## Author Contributions

All authors contributed and approved the completed manuscript. Manuscript drafting and statistics were completed by ER supervised by CL. Substantial edits were completed by CL, VM, and ES. Data were collected and study was organized and conceptualized by YL and SM, as part of YL’s masters thesis (Lee, 2014), supervised by ES.

## Conflict of Interest

The authors declare that the research was conducted in the absence of any commercial or financial relationships that could be construed as a potential conflict of interest.
