# Supplementary material for: Feedback-Related Negativity and Frontal Midline Theta Reflect Dissociable Processing of Reinforcement
Source: Front Hum Neurosci. 2020 Jan 9;13:452. doi: 10.3389/fnhum.2019.00452 (PMC6962175; doi:10.3389/fnhum.2019.00452)
Supplement: Supplementary file 1 [file Data_Sheet_1.docx]

**Supplementary Materials - Feedback-Related Negativity and Frontal Midline Theta Reflect Dissociable Processing of Reinforcement**

**S1.1 Statistical Analysis of Behavioral data, Delta, and Alpha Frequencies**

Behavioral differences between positive and negative reinforcement conditions were first examined using separate two-way 2 (positive or negative reinforcement) X 3 (low, medium, or high point level) repeated-measure ANOVAs on performance accuracy and reaction times. Because the control condition did not include multiple point levels, additional repeated-measure ANOVAs were conducted on performance accuracy and reaction times examining differences between positive reinforcement, negative reinforcement, and control trials. The three point levels were averaged over for these analyses (for positive and negative reinforcement).

Similarly, to determine if the observed ­task-related influences in alpha ranges were significant, alpha activity at FCz was analyzed in the same manner as neural activation in the main manuscript. Specifically, a 2 (positive or negative reinforcement) X 2 (correct or error) X 3 (low, medium, or high point level) repeated-measures ANOVA was conducted on alpha power. Since the control condition did not include multiple point levels, separate 3 (positive, negative, or no reinforcement) X 2 (correct or error) repeated-measures ANOVAs were conducted on alpha power. The three point levels were averaged over for these analyses for both positive and negative reinforcement. These same analyses were conducted on delta power at electrode Pz. Results of these analyses are presented in S1.4 and Supplementary Figure 2.

**S1.2 Alternative Analysis of Theta Power**

Since frontal midline is sometimes claimed to be primarily non-phase-locked, we computed theta power after subtraction of the ERP from single trials of data. All analyses were conducted identically to those in the main body. Specifically, a 2 (positive or negative reinforcement) X 2 (correct or error) X 3 (low, medium, or high point level) repeated-measures ANOVA was conducted on alpha power. Since the control condition did not include multiple point levels, separate 3 (positive, negative, or no reinforcement) X 2 (correct or error) repeated-measures ANOVAs were conducted on alpha power. The three point levels were averaged over for these analyses for both positive and negative reinforcement. Results of these analyses are presented in S1.5 and Supplementary Figure 3.

**S1.3 Behavior during positive and negative reinforcement task**

The initial ANOVA on performance accuracy indicated no effects of reinforcement type, point level, or their interaction. However, there was a main effect of point level on reaction times, *F*(2,100) = 4.35, *p* = .02, η^2^ = .08, ε = .91, which was subsumed by an interaction between reinforcement type and point level, *F*(2,100) = 3.10, *p* = .05, η^2^ = .06, ε = 1.0. At high point levels, reaction times were faster for positive reinforcement trials than for negative reinforcement, mean difference = 5.8 ms, 95% CI = [1.5, 10.10], *p* < .01. There were no differences between positive and negative reinforcement reaction times at medium or low point levels. In positive reinforcement trials, reaction times were significantly faster for high point trials than for both medium point trials, mean difference = 6.6 ms, 95% CI = [1.83, 11.35], *p* = .004, and low point trials, mean difference = 6.0 ms, 95% CI = [.62, 11.39], *p* = .02. Reaction times did not differ between medium and low point trials for positive reinforcement. In negative reinforcement trials, there were no reaction time differences between point levels. The second one-way repeated measures ANOVA indicated no effect of reinforcement type (positive, negative, or control) on performance accuracy or reaction times. See Supplemental Figure 1 for a summary of accuracy and reaction time results.

**S1.4 Condition Differences in Alpha & Delta Frequency Ranges**

The initial repeated-measures ANOVA on alpha power showed that the main effect of point value associated with the trial was not significant, nor were any of its interactions with other variables, ruling out point value as an explanation for any differences in alpha activation. The second ANOVA on alpha power indicated a main effect of outcome, *F*(1,50) = 6.67, *p* = .013, η^2^ = .12, ε = 1.0, such that error trials resulted in lower alpha activation than correct trials, mean difference = .20 dB, 95% CI = [.05, .36].

The initial repeated-measures ANOVA on delta power showed that the main effect of point value associated with the trial was not significant, nor were any of its interactions with other variables, ruling out point value as an explanation for any differences in delta activation. The second repeated-measures ANOVA on delta power indicated a main effect of outcome, *F*(1,50) = 6.81, *p* = .012, η^2^ = .12, ε = 1.0, such that error trials resulted in greater delta activation than correct trials, mean difference = .28 dB, 95% CI = [.06, .49]. Results of delta / alpha band analyses are depicted in Supplementary Figure 2.

**S1.5 Condition Differences in Non-phase-locked Theta Power**

The initial three-way repeated measures ANOVA showed no effect of point level (low, medium, or high) on FMΘ amplitude, *F*(1,100) = 1.21, *p* = .30, η^2^ = .02, ε = .93, and no interaction of point level with any other variables, all *p* > .2. These null results rule out point level as an explanation for any differences in theta activation. Therefore, low, medium, and high point level trials were averaged over for further theta analyses. A second two-way repeated measures ANOVA on FMΘ power indicated a main effect of outcome (correct or error), *F*(1,50) = 9.94, *p* = .003, η^2^ = .17, ε = 1.0, and a main effect of reinforcement type (positive, negative, or control), *F*(2,100) = 3.43, *p* = .045, η^2^ = .06, ε = .83. Post hoc linear contrasts (Section 2.4.3) were used to interpret the effects of reinforcement type and outcome. Results indicated that error trials resulted in greater theta activation than correct trials, mean difference = .38 dB, 95% CI = [.14, .63], *p* = .003. Positive reinforcement resulted in greater theta activation than control trials, mean difference = .27 dB, 95% CI = [.007, .52], *p* = .042.


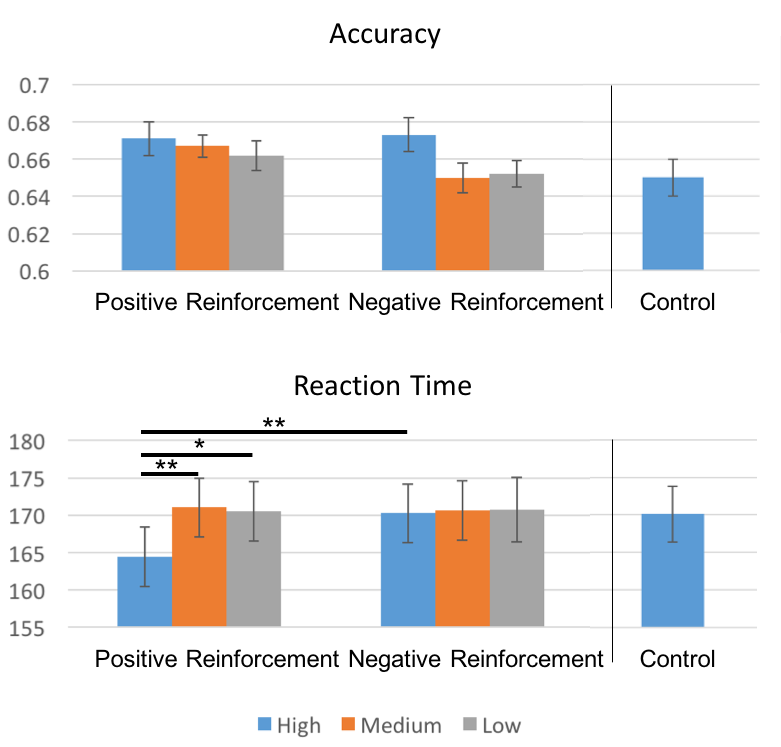


Supplementary Figure 1. Performance accuracy and reaction times across all trial types. * = *p* < .05, ** = *p* < .01. Error bars represent 1 +/- SEM.


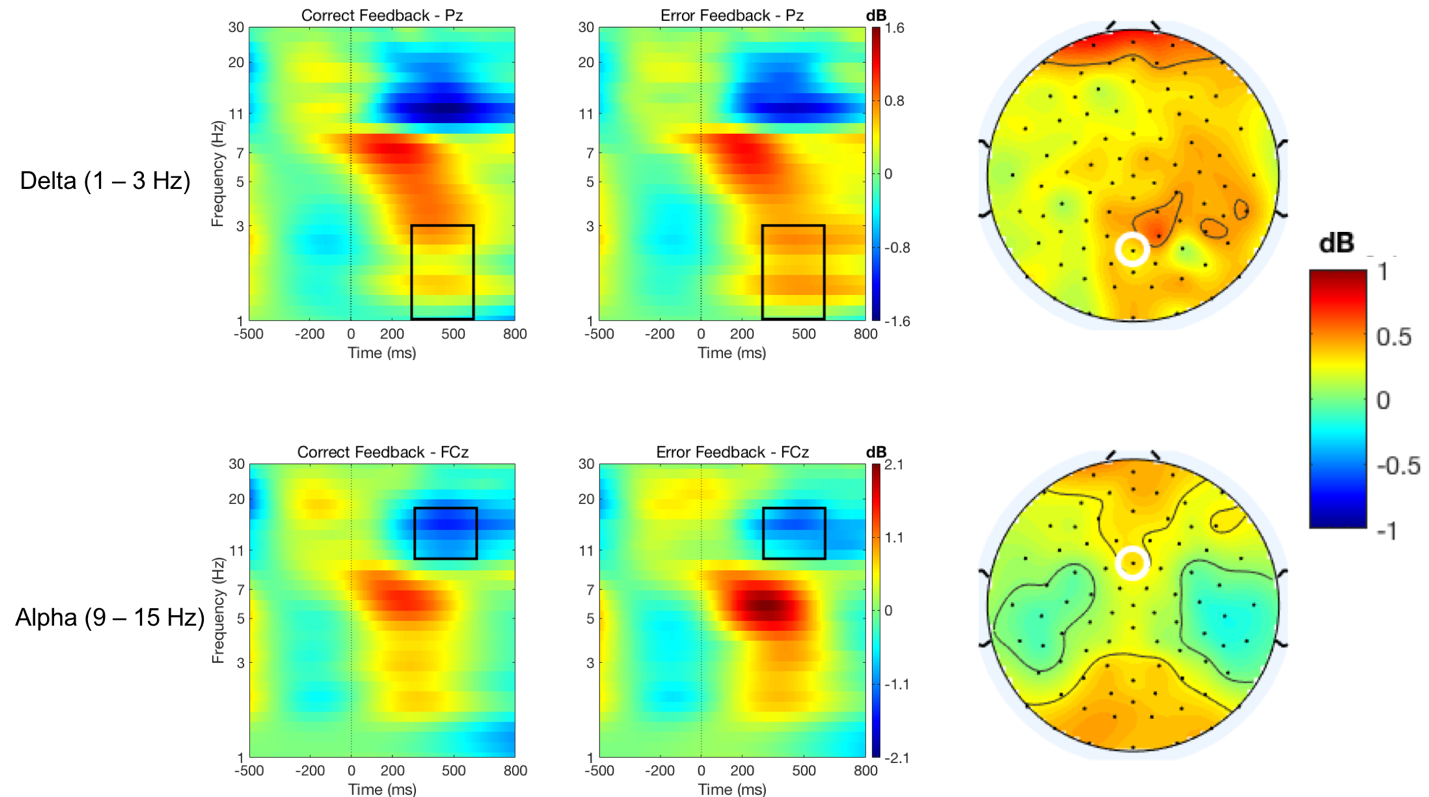


Supplementary Figure 2. Time-frequency spectrograms measured at Pz (top), and FCz (bottom). Topographic distributions indicate the error-correct difference in time X frequency ranges. Measurement electrode (Delta: Pz, Alpha: FCz) is marked by a white ellipse. Black outlines enclose the region of data exported for statistical analysis.


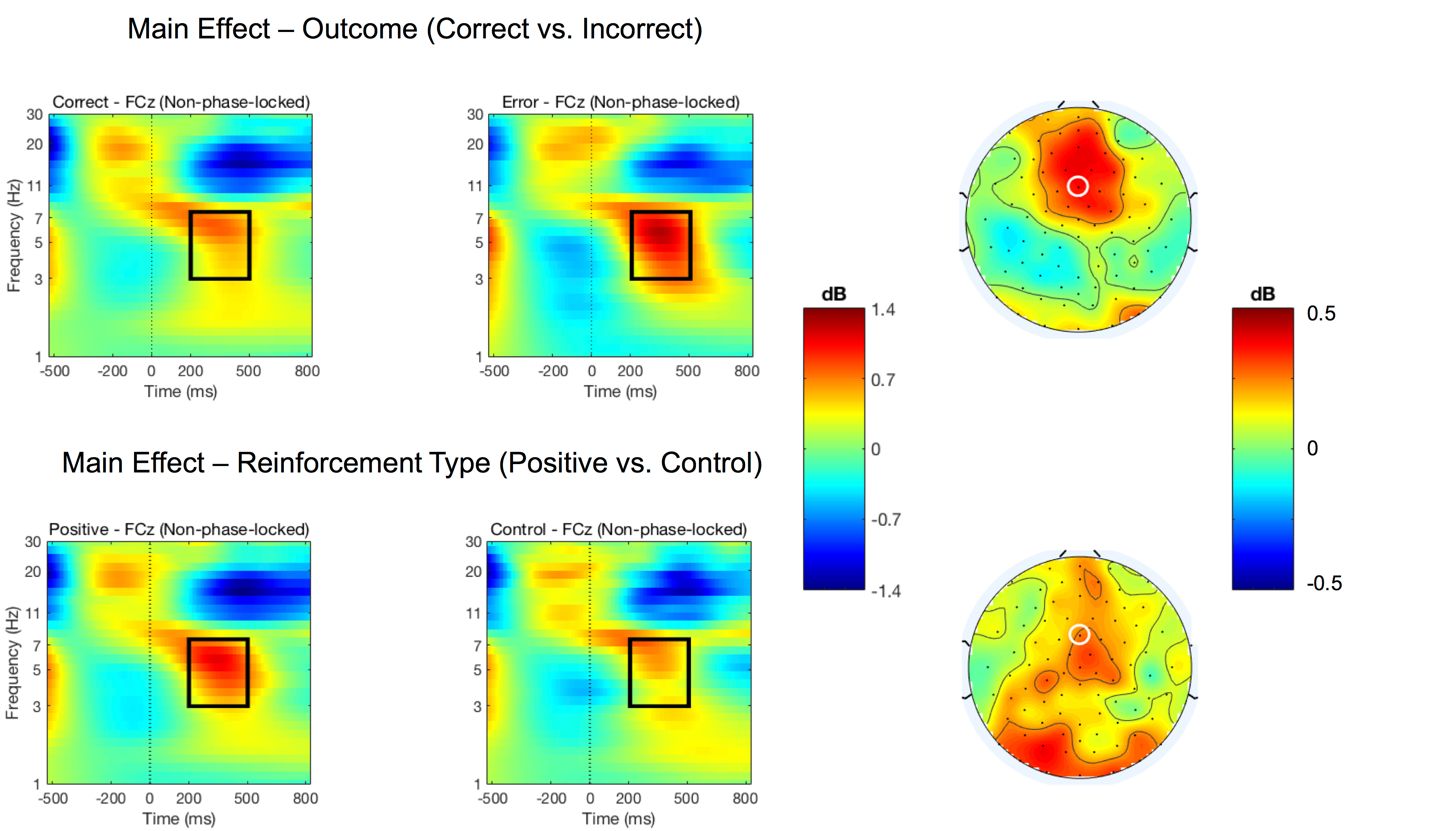


Supplementary Figure 3. Time-frequency spectrograms measured at FCz. ERSP is depicted following removal of ERP from single trials of EEG data. Top set of plots indicate the error-correct difference in time X frequency ranges. Bottom set of plots indicate significant difference between positive reinforcement and control trials. Measurement electrode (FCz) is marked by a white ellipse. Black outlines enclose the region of data exported for statistical analysis.


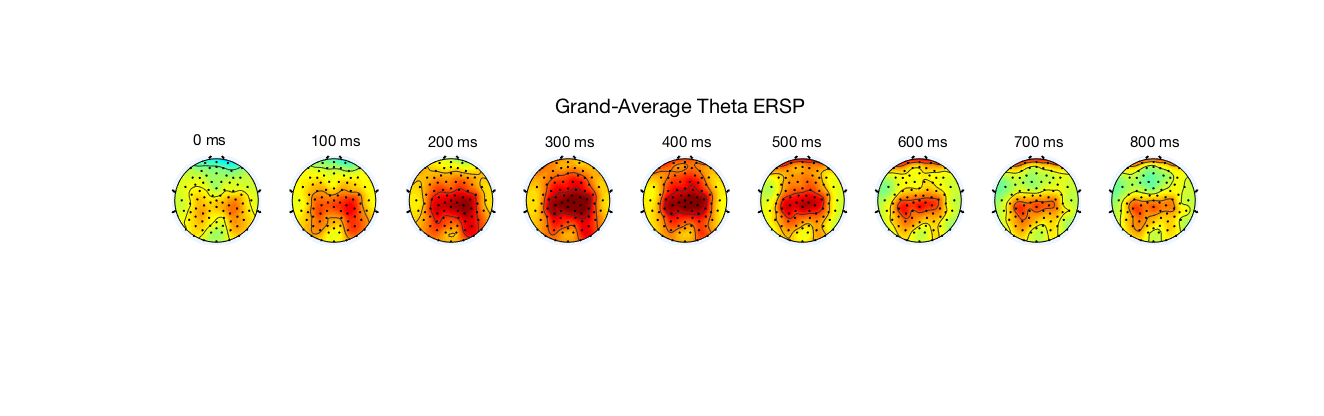


Supplementary Figure 4. Grand-averaged theta activity is maximal over medial and frontal sensors, and increases from roughly 200 – 450 ms at mediofrontal sensors.

Supplementary Table 1. Full output of first ANOVA on FRN amplitudes.

| Factor | *F* | *p* | η^2^ | ε |
| --- | --- | --- | --- | --- |
|  |  |  |  |  |
| Reinforcement Type | 1.0 | .32 | .02 | 1 |
|  |  |  |  |  |
| Point Level | 11.52 | 5e-4*** | .19 | .92 |
|  |  |  |  |  |
| Outcome | .73 | .40 | .01 | 1 |
|  |  |  |  |  |
| Reinforcement Type * Point Level | 1.66 | .20 | .03 | .94 |
|  |  |  |  |  |
| Reinforcement Type * Outcome | 50.50 | 4e-9*** | .50 | 1 |
|  |  |  |  |  |
| Point Level * Outcome | .50 | .61 | .01 | 1 |
|  |  |  |  |  |
| Reinforcement Type * Point Level * Outcome | .15 | .86 | .003 | .98 |

*** p < .001

Supplementary Table 2. Post hoc linear contrasts for interaction of reinforcement type X outcome from first ANOVA on FRN amplitudes.

| Reinforcement Type | Outcome | Mean | Difference | SE | *p* |
| --- | --- | --- | --- | --- | --- |
|  |  |  |  |  |  |
| Positive Reinforcement | Correct | 3.28 | .70 | .22 | .002** |
|  | Error | 2.58 |  |  |  |
|  |  |  |  |  |  |
| Negative Reinforcement | Correct | 2.33 | 1.01 | .21 | 2e-5*** |
|  | Error | 3.34 |  |  |  |
|  |  |  |  |  |  |

** p < .01, *** p < .001

Supplementary Table 3. Post hoc linear contrasts for interaction of reinforcement type X outcome from first ANOVA on FRN amplitudes.

| Outcome | Reinforcement Type | Mean | Difference | SE | *p* |
| --- | --- | --- | --- | --- | --- |
|  |  |  |  |  |  |
| Correct | Positive Reinforcement | 3.28 | .95 | .14 | 2e-8*** |
|  | Negative Reinforcement | 2.33 |  |  |  |
|  |  |  |  |  |  |
| Error | Positive Reinforcement | 2.58 | .75 | .17 | 4e-5*** |
|  | Negative Reinforcement | 3.34 |  |  |  |
|  |  |  |  |  |  |

*** p < .001

Supplementary Table 4. Full output of second ANOVA on FRN amplitudes.

| Factor | *F* | *p* | η^2^ | ε |
| --- | --- | --- | --- | --- |
|  |  |  |  |  |
| Reinforcement Type | 22.88 | 7e-9*** | .31 | 1 |
|  |  |  |  |  |
| Outcome | .33 | .57 | .01 | 1 |
|  |  |  |  |  |
| Reinforcement Type * Outcome | 17.14 | 1e-6*** | .26 | 1 |

*** p < .001

Supplementary Table 5. Post hoc linear contrasts for interaction of reinforcement type X outcome from second ANOVA on FRN amplitudes.

| Reinforcement Type | Outcome | Mean | Difference | SE | *p* |
| --- | --- | --- | --- | --- | --- |
|  |  |  |  |  |  |
| Positive Reinforcement | Correct | 3.28 | .70 | .22 | .002** |
|  | Error | 2.58 |  |  |  |
|  |  |  |  |  |  |
| Negative Reinforcement | Correct | 2.33 | 1.01 | .21 | 2e-5*** |
|  | Error | 3.34 |  |  |  |
|  |  |  |  |  |  |
| Control (no reinforcement) | Correct | 2.07 | .02 | .27 | .94 |
|  | Error | 2.05 |  |  |  |
|  |  |  |  |  |  |

** p < .01, *** p < .001

Supplementary Table 6. Post hoc linear contrasts for interaction of reinforcement type X outcome from second ANOVA on FRN amplitudes.

| Outcome | Reinforcement Type (I) | Reinforcement Type (J) | Difference | SE | *p* |
| --- | --- | --- | --- | --- | --- |
|  |  |  |  |  |  |
| Correct | Positive | Negative | .95 | .14 | 6e-8*** |
|  |  | Control | 1.22 | .19 | 1e-7*** |
|  | Negative | Control | .26 | .19 | .51 |
|  |  |  |  |  |  |
| Error | Positive | Negative | .75 | .17 | 1e-4*** |
|  |  | Control | .54 | .23 | .07 |
|  | Negative | Control | 1.29 | .28 | 7e-5*** |
|  |  |  |  |  |  |

*** p < .001

Supplementary Table 7. Full Output of First ANOVA on FMΘ power.

| Factor | *F* | *p* | η^2^ | ε |
| --- | --- | --- | --- | --- |
|  |  |  |  |  |
| Reinforcement Type | .61 | .44 | .01 | 1 |
|  |  |  |  |  |
| Point Level | 1.81 | .17 | .07 | .97 |
|  |  |  |  |  |
| Outcome | 16.67 | 1.6e-4*** | .25 | 1 |
|  |  |  |  |  |
| Reinforcement Type * Point Level | .32 | .72 | .01 | .95 |
|  |  |  |  |  |
| Reinforcement Type * Outcome | 15.01 | 3.1e-4*** | .23 | 1 |
|  |  |  |  |  |
| Point Level * Outcome | .65 | .50 | .01 | .86 |
|  |  |  |  |  |
| Reinforcement Type * Point Level * Outcome | .55 | .58 | .01 | 1 |
|  |  |  |  |  |

*** p < .001

Supplementary Table 8. Post hoc linear contrasts for interaction of reinforcement type X outcome from first ANOVA on FMΘ power.

| Reinforcement Type | Outcome | Mean | Difference | SE | *p* |
| --- | --- | --- | --- | --- | --- |
|  |  |  |  |  |  |
| Positive Reinforcement | Correct | .88 | .86 | .17 | 4e-6*** |
|  | Error | 1.74 |  |  |  |
|  |  |  |  |  |  |
| Negative Reinforcement | Correct | 1.18 | .13 | .14 | .38 |
|  | Error | 1.31 |  |  |  |
|  |  |  |  |  |  |

*** p < .001

Supplementary Table 9. Post hoc linear contrasts for interaction of reinforcement type X outcome from first ANOVA on FMΘ power.

| Outcome | Reinforcement Type | Mean | Difference | SE | *p* |
| --- | --- | --- | --- | --- | --- |
|  |  |  |  |  |  |
| Correct | Positive Reinforcement | .88 | .30 | .11 | .007** |
|  | Negative Reinforcement | 1.18 |  |  |  |
|  |  |  |  |  |  |
| Error | Positive Reinforcement | 1.74 | .44 | .15 | .004** |
|  | Negative Reinforcement | 1.31 |  |  |  |
|  |  |  |  |  |  |

** p < .01

Supplementary Table 10. Full output of second ANOVA on FMΘ power.

| Factor | *F* | *p* | η^2^ | ε |
| --- | --- | --- | --- | --- |
|  |  |  |  |  |
| Reinforcement Type | 1.50 | .23 | .06 | .84 |
|  |  |  |  |  |
| Outcome | 17.14 | 1e-4*** | .26 | 1 |
|  |  |  |  |  |
| Reinforcement Type * Outcome | 4.64 | .016* | .09 | .85 |

* p < .05, *** p < .001

Supplementary Table 11. Post hoc linear contrasts for interaction of reinforcement type X outcome from second ANOVA on FMΘ power.

| Reinforcement Type | Outcome | Mean | Difference | SE | *p* |
| --- | --- | --- | --- | --- | --- |
|  |  |  |  |  |  |
| Positive Reinforcement | Correct | .88 | .86 | .17 | 4e-6*** |
|  | Error | 1.74 |  |  |  |
|  |  |  |  |  |  |
| Negative Reinforcement | Correct | 1.18 | 1.01 | .21 | .38 |
|  | Error | 1.31 |  |  |  |
|  |  |  |  |  |  |
| Control (no reinforcement) | Correct | .78 | .64 | .26 | .016* |
|  | Error | 1.44 |  |  |  |
|  |  |  |  |  |  |

* p < .05, *** p < .001

Supplementary Table 12. Post hoc linear contrasts for interaction of reinforcement type X outcome from second ANOVA on FMΘ power.

| Outcome | Reinforcement Type (I) | Reinforcement Type (J) | Difference | SE | *p* |
| --- | --- | --- | --- | --- | --- |
|  |  |  |  |  |  |
| Correct | Positive | Negative | .30 | .11 | .021* |
|  |  | Control | .09 | .19 | 1 |
|  | Negative | Control | .39 | .19 | .14 |
|  |  |  |  |  |  |
| Error | Positive | Negative | .44 | .15 | .013* |
|  |  | Control | .31 | .17 | .23 |
|  | Negative | Control | .13 | .19 | 1 |
|  |  |  |  |  |  |

* p < .05
